# Fatal pneumonitis associated with postoperative intensity-modulated radiotherapy in lung cancer: Case report and review

**DOI:** 10.3892/ol.2012.1053

**Published:** 2012-11-30

**Authors:** YAN HU, JINGJING LI, XIAOYAN SU

**Affiliations:** Department of Oncology, The First People’s Hospital of Jingzhou, Jingzhou, Hubei 434000, P.R. China

**Keywords:** radiation pneumonitis, intensity-modulated radiotherapy, low-dose irradiated volume

## Abstract

Radiation pneumonitis (RP) is the most significant complication of acute treatment-related toxicities in lung cancer. Intensity-modulated radiotherapy (IMRT) with inverse planning enables us to achieve the desired dose distribution. However, there are many high-risk procedures associated with lung irradiation, including chemotherapy and surgery. We report a case of fatal treatment-related pneumonitis, where the patient had undergone postoperative IMRT for lung cancer. Following completion of radiotherapy, the patient developed progressive dyspnea. A chest computed tomography (CT) scan revealed the presence of diffuse reticular interstitial processes and honeycombing in both lungs. The fibrotic change in both lungs in a transverse view was compatible with low-dose irradiation of non-target organs at risk. Acute radiation pneumonitis was diagnosed. For patients receiving postoperative IMRT, low-dose irradiation volumes should be considered for lungs, as well as strict dose-volume histogram (DVH) parameters.

## Introduction

Due to advances in radiation technology, intensity-modulated radiotherapy (IMRT) has been widely applied in cancer treatment. IMRT with inverse planning enables us to achieve the desired dose distribution, due to its ability to provide sharp dose gradients at the junction of the tumor and the adjacent critical organs (1). Although it is considered safe and effective, IMRT should be regarded with caution; lung irradiation involves high-risk procedures, including chemotherapy and surgery. Here, we report a case of fatal treatment-related pneumonitis caused by postoperative IMRT in lung cancer. The study was approved by the Ethics Xommittee of the First People’s Hospital of Jingzhou, Jingzhou, Hubei, China. Written informed patient consent was obtained from the patient’s family.

## Case report

A 65-year-old male patient presented with a dry cough for one month, and a computed tomography (CT) scan revealed a mass in the right upper lobe of the lung. The patient was referred to the First People’s Hospital of Jingzhou (Jingzhou, China) for detailed examination and treatment. The patient underwent a right upper lobectomy on 6th January, 2011. The postoperative pathological diagnosis confirmed the mass to be a squamous cell carcinoma (pT2N2M0, stage IIIA). The lymph node metastasis was also pathologically diagnosed as a squamous cell carcinoma.

Three weeks post-surgery, the patient received adjuvant chemotherapy with cisplatin and vinorelbine. One week after two complete cycles of chemotherapy, IMRT was delivered using the Eclipse treatment planning system with a 6 MV linear Varian-23EX accelerator (Varian Medical Systems, Inc., Palo Alto, CA, USA).

Surgical stump, ipsilateral hilar and ipsilateral mediastinal lymph node were delineated as the clinical target volume (CTV). The planning target volume (PTV) was defined as the CTV with a 6-mm margin for tumor motion and set-up uncertainty. The contoured organs at risk (OARs), dose constraints/penalty functions and planning parameters are listed in Tables I and II. IMRT was delivered at a dose of 50.4 Gy (1.8 Gy/fraction).

**Figure 1.**
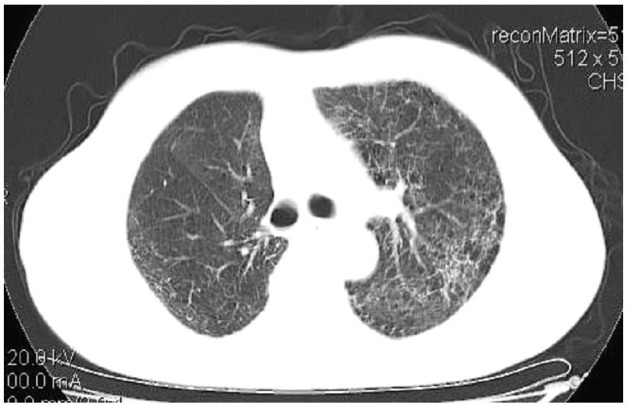
Chest computed tomography (CT) scan.

**Figure 2.**
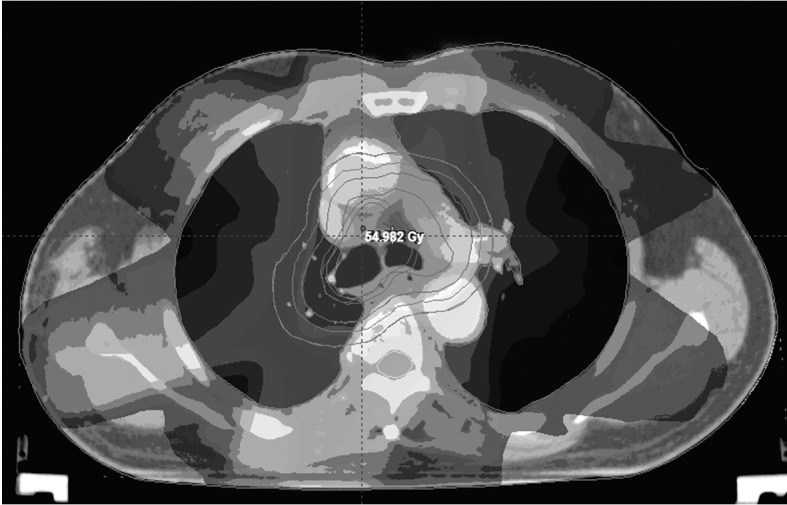
The dose distribution of radiotherapy designed for intensity-modulated radiotherapy (IMRT).

Radiotherapy was completed on 28th March. However, the patient developed a low-grade fever, a dry cough and dyspnea on 30th March. A chest CT scan demonstrated the presence of diffuse reticular interstitial processes and honeycombing in both lungs (Fig. 1). An atypical infection was suspected. The blood and sputum culture results were negative, and the fibrotic change in both lungs in a transverse view was compatible with low-dose irradiation of non-target OARs (Figs. 1 and 2). Acute radiation pneumonitis (RP) was diagnosed and empirical antibiotics were injected. Steroid therapy comprising methylprednisolone (40 mg), 20 mg iv. q8h, was administered for inflammatory lung disease. The patient also received supportive treatment simultaneously. However, the patient succumbed due to respiratory failure after one week.

## Discussion

RP is a common complication in radiotherapy and is the most significant treatment-related toxicity in lung cancer. The symptoms of treatment-related pneumonitis (TRP) are a dry cough, a low-grade fever, chest pain and shortness of breath. Such clinical symptoms of RP may lead to a poor quality of life for lung cancer patients and even induce fatality. The MD Anderson experience demonstrated that the rates of grade ≥3 maximum treatment-related pneumonitis (TRP max) were 11 and 14% at 6 and 12 months, respectively. IMRT has been observed to offer TRP rates that are comparable with those achieved by three-dimensional conformal radiotherapy (3D-CRT) (2).

The majority of DVH parameters have been demonstrated to be associated with the occurrence of RP (14). The incidence and grade of RP have been revealed to be significantly correlated with the V20 value (percentage of both lungs receiving >20 Gy, not including the gross tumor volume) and mean lung dose (MLD) (3). Thus, the V20 value is hypothesized to be a factor that may be used for predicting RP after concurrent chemoradiation for lung cancer. Kong *et al*(4) demonstrated that the V20 value should be <35% in 3D-CRT. Belderbos *et al*(5) revealed that the overall treatment time is safe for small-volume lung tumors with an MLD ≤13.6 Gy. In the present case, the V20 value was 27.5% and the MLD was 12.6 Gy; both of which are lower than the recommended value. However, the V5 value (72.4%) was higher than the recommended value. Therefore, it is possible that the low-dose irradiated volume induced the fatal pneumonitis.

IMRT delivered in a static mode produces homogeneous dose distributions in the target. However, Murshed *et al*(6) demonstrated that when dynamic multi-leaf collimator (MLC) sequencing was selected, the increased volume of lung irradiated at 5 Gy was observed to be a potential problem when IMRT was applied in the lung region. In a study by Yorke *et al*(7), the V5, V10 and V13 values for total and ipsilateral lung were more strongly correlated than the lung figures currently used in clinical anlaysis (total lung V20, mean dose, effective uniform dose, normal tissue complication probability and fraction of damaged subunits). The MD Anderson experience (2,8) also revealed that the V10 and V20 values of the normal lung decreased by a median of 7 and 10%, respectively, with IMRT. However, the V5 value of thoracic tissue increased with IMRT, and rV5 was the only factor to be significantly correlated with lung toxicity.

Postoperative radiotherapy is capable of significantly improving the survival of patients with resected pathological stage IIIA-N2 non-small cell lung cancer (NSCLC) (9). For patients receiving postoperative radiotherapy, the appropriate dose limits are not yet clear. Ji *et al*(10) demonstrated that >340 cm^3^ of the ipsilateral lung receiving 30 Gy was significantly correlated with the risk of RP in patients undergoing a lobectomy. It was considered safe for patients who undergo a pneumonectomy to receive postoperative 3D-CRT, provided that the lung V20 value was <10%.

IMRT planning is dependent on the treatment-planning systems (TPSs) and MLC, while data in low-dose regions is often ignored. Jang *et al*(11) found that in the commercial TPS-generated IMRT plans, dose calculation errors primarily occurred in the low-dose regions of IMRT plans (<50% of the radiation dose prescribed for the tumor). These errors were identified to be caused by inadequate modeling of the MLC transmission and leaf scatter in commercial TPSs.

In addition, there are different views concerning concurrent or sequential chemoradiotherapy-related RP. Shi *et al*(12) demonstrated that thoracic concurrent chemoradiotherapy should be planned with caution with IMRT when the volume of normal lung receiving ≥10 Gy is large. A study by Vogelius *et al*(13) demonstrated that for radiotherapy alone, the critical volume model predicts that the two IMRT plans (TPS and MLC) are associated with a lower risk of radiation pneumonitis than the 3D-CRT plan. However, when the chemotherapy equivalent radiation dose exceeds a certain threshold, the radiation pneumonitis risk following IMRT is greater than that which follows 3D-CRT. The threshold dose is within the range estimated from clinical chemoradiotherapy data sets (13).

Treatment of lung cancer remains a significant challenge for modern medicine. It is essential to evaluate the DVH of critical structures and to limit the doses to lungs. The curve composed of multiple DVH parameters was demonstrated to be more important than any single DVH parameter (14). For patients receiving postoperative IMRT, low-dose irradiation volumes should be considered for lungs, as well as strict DVH parameters.

## Figures and Tables

**Table I.** Dose/volume constraints and mean dose for sensitive structures.

Structure	Importance	Max. dose constraint (Gy)	Max. volume constraint (%)	Mean dose (Gy)
Heart	50	50	50	13.2
Lungs	70	17	22	12.6
Spinal cord	70	35	42	15.6

**Table II.** Lung planning parameters.

Parameters	Left lung	Right lung	Both lungs
V30 (%)	4.7	34.4	17.0
V20 (%)	9.6	52.7	27.5
V10 (%)	32.7	79.28	52.0
V5 (%)	64.0	84.5	72.4
Mean dose (Gy)	9.4	24.3	12.6
